# Vitamin D Status and Reproductive Hormonal Profiles in Early Versus Physiological Menopause: A Comparative Observational Study

**DOI:** 10.3390/biomedicines14061283

**Published:** 2026-06-04

**Authors:** Anamaria Ardelean, Cristian Furău, Oana Toduț, Nicoleta Mirica, Florina Buleu, Simona Ioana Sipos, Ion Petre, Izabella Petre, Tiberiu Buleu, Mircea Iurciuc, Oana Suciu, Roxana Furău

**Affiliations:** 1 Multidisciplinary Doctoral School, “Vasile Goldis” Western University of Arad, 310414 Arad, Romania; ardelean.anamaria@uvvg.ro (A.A.); furau.cristian@uvvg.ro (C.F.); todut.oana@uvvg.ro (O.T.); 2 Department of Life Sciences, “Vasile Goldis” Western University of Arad, 310414 Arad, Romania; 3 Center for Translational Research and Systems Medicine, “Victor Babes” University of Medicine and Pharmacy, 300041 Timisoara, Romania; mirica.nicoleta@umft.ro; 4 Department of Kinetotherapy and Motor Skills, Faculty of Physical Education and Sport, West University of Timișoara, 300223 Timisoara, Romania; 5 Department of Cardiology, “Victor Babes” University of Medicine and Pharmacy, Eftimie Murgu Square 2, 300041 Timisoara, Romania; 6 County Emergency Clinical Hospital “Pius Brinzeu”, 300732 Timisoara, Romania; petre.izabella@umft.ro (I.P.); tiberiu.buleu@umft.ro (T.B.); 7 Department of Biochemistry and Pharmacology, “Victor Babes” University of Medicine and Pharmacy Timisoara, Eftimie Murgu Square 2, 300041 Timisoara, Romania; 8 Department of Functional Sciences, Medical Informatics and Biostatistics Discipline, “Victor Babes” University of Medicine and Pharmacy, 300041 Timisoara, Romania; petre.ion@umft.ro; 9 Department of Obstetrics and Gynaecology, “Victor Babes” University of Medicine and Pharmacy, 300041 Timisoara, Romania; 10 Faculty of General Medicine, “Victor Babes” University of Medicine and Pharmacy, 300041 Timisoara, Romania; 11 Department of Clinic Nursing, “Victor Babes” University of Medicine and Pharmacy, Eftimie Murgu Square 2, 300041 Timisoara, Romania; iurciuc.mircea@umft.ro; 12 Department of Microbiology, “Victor Babes” University of Medicine and Pharmacy, Eftimie Murgu Square 2, 300041 Timisoara, Romania; oana.suciu@umft.ro; 13 Department of General Medicine, Faculty of Medicine, “Vasile Goldis” Western University of Arad, 310414 Arad, Romania; furau.roxana@uvvg.ro

**Keywords:** early menopause, vitamin D, estradiol, progesterone, ovarian insufficiency, follicle-stimulating hormone, luteinizing hormone

## Abstract

**Background:** An early menopause (by definition, menopause that occurs at a woman’s age 40 through 45) is often associated with certain changes in the body that can result in risks for health-related conditions, an extended period later. Thus, scientists have begun examining how vitamin D has been suggested to be associated with endocrine function regulating both hormones and reproductive function during this time. However, it is not yet clear as to whether or not vitamin D provides any benefit to women who have experienced an early menopause. **Material and Methods:** The data was collected from 272 women in this retrospective, observational study at The County Hospital, Department of Obstetrics and Gynecology, Arad. The method of grouping the sample included two stratifications into early and physiological menopause categories based on amenorrhoea for a minimum of 12 consecutive months. 25-hydroxyvitamin D (25(OH)D) levels were classified into three categories: deficiency (<20 ng/mL), insufficiency (21–29 ng/mL), or adequacy (≥30 ng/mL). Estradiol, progesterone, follicle-stimulating hormone (FSH), and luteinizing hormone (LH) hormone parameters were measured using standard immunoassays. The analysis employed correlation and regression to evaluate potential relationships between 25(OH)D levels and hormone parameters. **Results:** A significant proportion of the study group had a vitamin D deficiency. This was supported by the fact that only 24.27% of women were identified as having adequate levels of vitamin D, while the rest (62.03%) did not. Women in the early menopause group had a statistically significant negative relationship between estradiol and FSH (i.e., r = −0.29, *p* = 0.0016), as well as between progesterone and LH (i.e., r = −0.207, *p* = 0.026). There was not a statistically significant relationship between total sample vitamin D and estradiol (i.e., r = −0.038, *p* = 0.686) nor between vitamin D and progesterone (i.e., r = 0.031, *p* = 0.744). Women with vitamin D blood levels of 30 ng/mL or more showed a strong negative relationship between vitamin D and estradiol (r = −0.780; *p* = 0.0016) and a moderate positive relationship with progesterone (r = 0.534; *p* = 0.0104). However, these relationships were inconsistent in other groups. All group comparative analyses showed that women in the early menopause group had much lower estradiol levels than those in the physiological menopause group, regardless of whether they were classified based on their vitamin D levels (*p* < 0.0001). **Conclusions:** Women experiencing early or physiological menopause are at risk of having low vitamin D levels. However, our study results do not show a consistent relationship between serum 25(OH)D concentrations and serum estradiol or progesterone concentrations among the study population, suggesting that vitamin D is not a major factor influencing hormonal changes during menopause. These findings were inconsistent across analyses and should be interpreted cautiously. Overall, the results do not support a significant association between serum 25(OH)D concentrations and reproductive hormone levels in our study population.

## 1. Introduction

The menopause is the natural manifestation of a biological process that leads to the complete cessation of the menstrual cycle in women, as a result of declining hormone levels. This can be confirmed by the absence of menstruation (amenorrhea) for twelve months [1]. Most women around the globe will naturally go through menopause between the ages of 40 and 60, although the exact age varies depending on various factors, such as standard of living and geographic location [2]. Women living in wealthy regions typically begin to go through menopause at an average age of 50–51, while women living in poorer countries typically go through menopause at a younger average age [2,3].

During the transition to menopause, women experience a range of symptoms and physiological changes. Among other symptoms, approximately 50% to over 80% of women report experiencing hot flashes and night sweats (vasomotor symptoms), as well as sleep disturbances, mood swings, or cognitive problems; these latter symptoms of depression, anxiety, or worry have been reported by 60–70% of women [4,5]. Throughout this transition, hormone levels fluctuate, often causing a woman to experience cognitive difficulties, including impaired concentration and memory difficulties with focus and/or reduced cognitive performance and concentration difficulties, with approximately 60% of women being adversely affected because of those changes [6]. These changes may substantially affect quality of life and long-term health outcomes in aging women. Recent literature has additionally emphasized the multifactorial endocrine and metabolic adaptations occurring during the menopausal transition and the importance of identifying modifiable factors potentially associated with women’s health during this period [7].

In addition to the onset of symptoms, menopause also leads to significant health risks throughout life. Lower estrogen levels dramatically increase a woman’s risk for losing bone density, developing cardiovascular disease, and experiencing alterations in metabolic and gastrointestinal function, and contribute to decreased cognitive function [8]. Women experiencing early menopause are at an even greater risk since their estrogen levels are low for an extended period of time. Therefore, these women are at greater risk for developing osteoporosis and heart disease, and dying from both of these diseases, than women who go through menopause at a middle age [9].

There is growing interest in how vitamin D can improve quality of life during menopause by contributing to overall health and well-being. Vitamin D is essential for maintaining calcium balance in the body and strengthening bones; it works in tandem with estrogen, contributing to bone remodeling and calcium homeostasis. After estrogen levels drop during menopause, women may experience reduced calcium absorption associated with estrogen deficiency. Women with vitamin D levels lower than normal are at a higher risk of developing osteoporosis [10,11].

Vitamin D has various effects that go beyond simply contributing to calcium absorption in the bones. This is supported by studies showing that vitamin D receptors are located in many bodily systems, including the ovaries, endometrium, and hypothalamic-pituitary axis, suggesting that vitamin D may influence hormones [12,13]. Overall, data obtained from research suggest that vitamin D has been proposed to be associated with processes related to ovarian aging through its influence on ovarian steroid production, follicle development, and expression of anti-Müllerian hormone [14].

Women at all stages of their reproductive lives are frequently diagnosed with vitamin D deficiency [15,16], particularly during menopause. Vitamin D deficiency is associated with the severity of certain menopause-related symptoms (e.g., musculoskeletal pain, fatigue, mood disorders, and overall quality of life) [17,18]. Some of this evidence suggests that women with low vitamin D levels may have a diminished ovarian reserve and may experience an earlier onset of menopause, partly due to low vitamin D levels. However, these relationships between low vitamin D levels and ovarian reserve or early menopause are not currently clearly defined [19].

Although there has been growing interest in this area of research, the available information regarding the relationship between reproductive hormones and vitamin D remains limited. Some researchers have indicated that they have found associations between estradiol or progesterone levels and vitamin D levels [20,21,22]. Nevertheless, most of the available studies have been conducted primarily on either women of childbearing age or postmenopausal women, and there is very little research on this topic specifically in women with early menopause as an independent group. The literature on early menopause is lacking, as no research has been conducted on early menopause. Early menopause has been hypothesized to present distinct clinical characteristics; however, its endocrine profile remains insufficiently characterized. How vitamin D levels affect the hormonal profile of this group remains unknown. Therefore, the aim of this study is to perform a comparative observational analysis of the relationship between serum vitamin D levels and reproductive hormones in women with early versus physiological menopause. Rather than addressing mechanistic pathways, this study seeks to clarify whether consistent associations exist within these populations and to contextualize these findings within the existing body of epidemiological evidence.

## 2. Materials and Methods

### 2.1. Population and Study Design

The study was conducted over a two-year period and included women admitted to the Department of Obstetrics and Gynecology at the County Hospital in Arad, Romania. All participants were of Romanian origin and had been experiencing secondary amenorrhea for at least 12 months. Women using vitamin D supplements, calcium supplements, multivitamins, or hormone replacement therapy were excluded, as these can suppress the hypothalamic–pituitary–ovarian axis and interfere with the accurate assessment of endogenous hormone levels.

**Figure 1:**
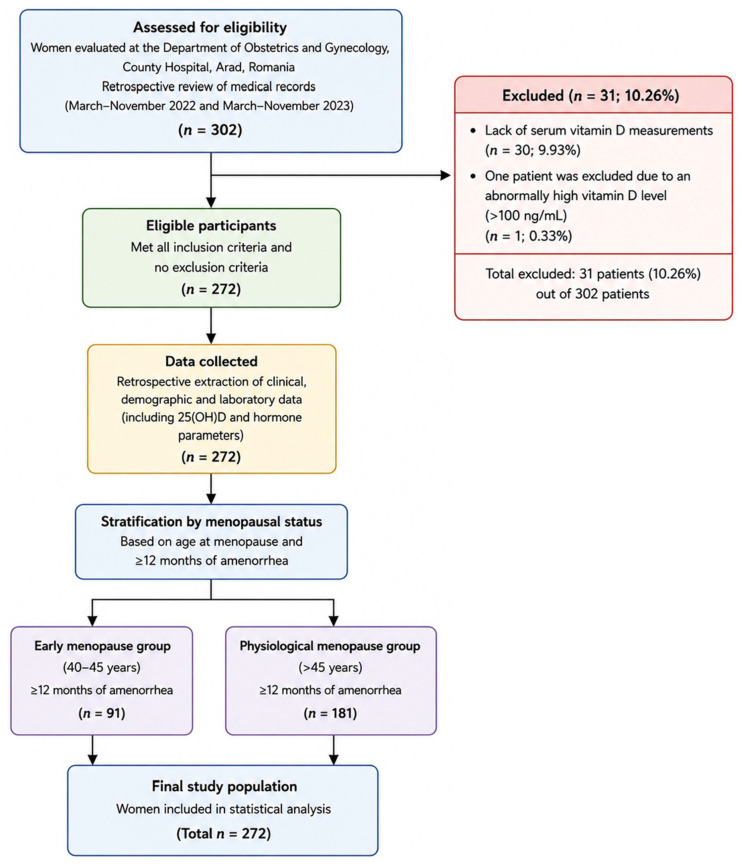
Study flowchart of patient selection and classification.

A total of 302 patients were initially identified. Of these, 10.26% were excluded due to the lack of serum vitamin D measurements, and one patient was excluded due to an abnormally high vitamin D level (>100 ng/mL), which was considered biologically atypical and potentially related to undocumented supplementation, laboratory variability, or other unmeasured factors. The final study population consisted of 272 women included in the analysis. The patient selection process and study design are summarized in the study flowchart (Figure 1).

The diagnosis of early menopause (defined as onset between the ages of 40 and 45) was established based on both clinical and laboratory criteria, in accordance with current clinical practice guidelines [23]. Clinically, early menopause was defined by the presence of amenorrhea or oligomenorrhea that persisted for at least four consecutive months, confirmed by clinical evaluation. Biochemically, menopause was confirmed by elevated levels of follicle-stimulating hormone (N.V.: >25–40 IU/L) measured in two separate blood samples collected at least 4–6 weeks apart, in accordance with laboratory reference ranges. This hormonal profile reflects reduced ovarian estrogen production and compensatory pituitary hypersecretion of gonadotropins, including FSH and luteinizing hormone LH [23].

### 2.2. 25-Hydroxyvitamin D Analysis

Serum 25(OH)D levels were determined using the DIAsource 25-OH Vitamin D Total ELISA kit (DIAsource Immunoassays SA, Louvain-la-Neuve, Belgium), following the manufacturer’s instructions. Analytical performance was assessed using low- and high-concentration control samples, tested in duplicate across 10 separate runs to evaluate reproducibility, and within a single run to assess repeatability. Calibration and validation were performed using the Free 25OH Vitamin D serum control provided by the manufacturer.

The limit of blank (LOB) was 2.012 ng/mL, while the limit of detection (LOD) was calculated at 3.035 ng/mL. Serum vitamin D status was classified as deficiency (<20 ng/mL), insufficiency (21–29 ng/mL), or sufficiency (≥30 ng/mL), according to guideline recommendations [24].

None of the participants reported using vitamin D, calcium, or other micronutrient supplements or multivitamins, and none were undergoing hormone replacement therapy. To minimize seasonal variability in serum 25(OH)D concentrations, only women whose blood samples were collected between 1 March and 1 November 2022–2023 were included. Additionally, based on medical history, none of the participants reported excessive sun exposure prior to sampling.

### 2.3. Hormonal Assessment

Serum estradiol, progesterone, follicle-stimulating hormone, and luteinizing hormone concentrations were measured using automated chemiluminescent immunoassays performed in the hospital’s certified clinical laboratory according to manufacturer protocols and standardized laboratory procedures. Internal quality control and calibration procedures were routinely performed in accordance with laboratory standards to ensure analytical reliability. Blood samples were collected and processed under standardized conditions to ensure analytical accuracy and reproducibility.

Hormonal values were interpreted according to laboratory-specific reference ranges. Postmenopausal status was characterized by elevated FSH levels (25.8–134.8 mIU/mL) and increased LH levels (7.7–58.5 mIU/mL), along with reduced estradiol concentrations (5–54.7 pg/mL) and low progesterone levels (<0.05–0.126 ng/mL). These reference intervals reflect the endocrine changes associated with ovarian insufficiency and were used to confirm menopausal status in the study population.

### 2.4. Ethical Considerations

The study was conducted in accordance with the ethical principles outlined in the Declaration of Helsinki. Ethical approval was obtained from the Ethics Committee of Arad County Hospital (approval number: 32/05.06.2024). All participants signed informed consent forms before being included in the study, and data confidentiality and anonymity were strictly maintained throughout the research process.

### 2.5. Statistical Analysis

Statistical analyses were performed to evaluate the relationship between serum vitamin D status and hormonal parameters in women with early and physiological menopause. All analyses were carried out using JASP (Version 0.96.0; JASP Team, 2026), and a two-tailed *p*-value < 0.05 was considered statistically significant.

The demographic and clinical characteristics of the study population were summarized using descriptive statistical methods. Continuous variables were reported as mean ± standard deviation for normally distributed data, or as median with interquartile range when normality assumptions were not met. Categorical variables were expressed as frequencies and percentages. Only complete cases were included in the analyses.

The distribution of the data was assessed using the Shapiro–Wilk test and, based on these results, appropriate parametric or non-parametric statistical methods were applied.

Correlation analyses were conducted to examine the association between vitamin D status and hormonal parameters, including estradiol, progesterone, FSH, and LH. Depending on data distribution, either Pearson’s or Spearman’s correlation coefficients were applied.

Linear regression analysis was used to assess the potential influence of vitamin D status on hormonal parameters. In these models, vitamin D status was considered the independent variable, while estradiol and progesterone were included as dependent variables. Results were reported using regression coefficients, 95% confidence intervals, and R^2^ values.

Comparative analyses between early and physiological menopause groups, as well as across vitamin D subgroups, were performed using appropriate statistical tests depending on data distribution. For comparisons between two independent groups, independent samples *t*-tests were used for normally distributed variables, while the Mann–Whitney U test was applied for non-normally distributed data. For comparisons involving more than two groups, one-way ANOVA or the Kruskal–Wallis test was used, as appropriate.

All results were interpreted in the context of both statistical significance and clinical relevance. No adjustments for multiple comparisons were applied, and missing data were handled using complete-case analysis.

## 3. Results

### 3.1. Distribution of Vitamin D Levels and Menopause Categories

The distribution of female participants by menopause category and vitamin D level is presented in Table 1. The early menopause group (aged 40–45 years) included 91 subjects, while the physiological menopause group (aged 46–55 years) represented the majority of the study population (*n* = 181). A smaller proportion of participants were younger than 40 years (*n* = 28) and older than 55 years (*n* = 11).

Overall, vitamin D deficiency (<20 ng/mL) was identified in 38.97% of participants, insufficiency (21–29 ng/mL) in 36.76%, and sufficiency (≥30 ng/mL) in 24.27%.

Comparative analysis between early and physiological menopause indicates that, apart from age, no statistically significant differences were observed in hormonal or vitamin D parameters between the group with early menopause and the group with physiological menopause. Although numerically higher gonadotropin levels (FSH and LH) and lower progesterone concentrations were observed in the physiological menopause group, these differences did not reach statistical significance, indicating substantial overlap in endocrine profiles between the two groups. Several hormonal variables demonstrated substantial variability and skewed distributions. Although mean vitamin D values appeared numerically different between groups, median values and interquartile ranges demonstrated considerable overlap, consistent with the absence of statistically significant differences in serum 25(OH)D concentrations between menopause categories (*p* = 0.299). These findings are consistent with the absence of a measurable association between vitamin D status and reproductive hormone parameters in this cohort (Table 2).

**Figure 2:**
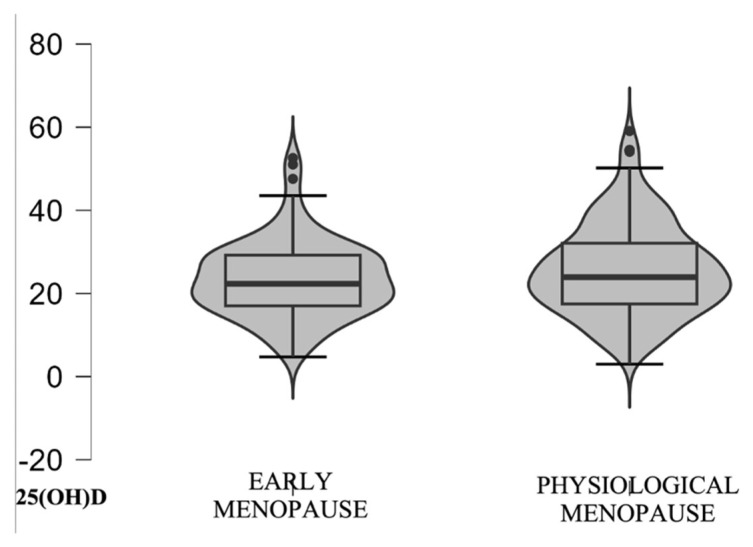
Serum 25(OH)D levels by menopause category. Violin plots with embedded box-and-whisker plots illustrate the distribution of serum 25(OH)D concentrations in women with early menopause and physiological menopause. The central line indicates the median, the box represents the interquartile range, and the “violin” shape reflects the density of the data distribution (*p* = 0.299).

The distribution of serum 25(OH)D levels between the early menopause group and the physiological menopause group is illustrated in Figure 2. Both groups exhibited similar distribution patterns, with a significant overlap in median values and interquartile ranges. Although slightly higher median vitamin D levels were observed in the group with physiological menopause, the difference was not statistically significant (*p* = 0.299), consistent with the results presented in Table 3.

### 3.2. Hormonal Profile in Early Menopause Group (n = 91)

In the early menopause group, the mean age of the participants was 41.08 ± 4.38 years, with a median of 42 years. Hormonal parameters showed marked variability, reflecting the heterogeneity of endocrine function within this cohort. Mean estradiol levels were 90.00 ± 108.66 pg/mL, while FSH and LH levels averaged 33.63 ± 37.41 mIU/mL and 18.68 ± 16.56 mIU/mL, respectively. Progesterone levels were generally low (1.66 ± 3.75 ng/mL), consistent with impaired ovarian function.

Serum levels of 25-hydroxyvitamin D [25(OH)D] also showed a wide distribution, with a mean of 36.33 ± 13.01 ng/mL. However, the median value (22.23 ng/mL) indicates that a considerable proportion of participants had insufficient or deficient vitamin D levels, suggesting an asymmetric distribution with higher outliers (Table 3).

The correlation analysis revealed a weak but statistically significant inverse relationship between estradiol and FSH levels (r = –0.29, *p* = 0.0016), indicating that lower estradiol concentrations are associated with higher gonadotropin levels. Similarly, progesterone showed a weak negative correlation with LH (r = –0.207, *p* = 0.026).

No statistically significant associations were identified between vitamin D levels and estradiol or progesterone, suggesting that 25(OH)D does not directly influence the hormonal profile in early menopause. These findings are summarized in Table 4.

Subgroup analyses stratified by vitamin D levels did not reveal any statistically significant associations between vitamin D and hormonal parameters in participants with deficient (<20 ng/mL) or insufficient (21–29 ng/mL) levels. Specifically, correlation analyses conducted in these subgroups did not reveal significant relationships between vitamin D and estradiol or progesterone. In contrast, among participants with sufficient vitamin D levels (≥30 ng/mL), a strong inverse correlation was observed between vitamin D and estradiol (r = −0.780, *p* = 0.0016), along with a moderate positive correlation with progesterone (r = 0.534, *p* = 0.0104). However, these findings were not consistent across subgroups and are likely influenced by the small sample size and statistical variability; therefore, they should be interpreted with caution (Table 5).

Linear regression analysis did not demonstrate a significant effect of vitamin D levels on estradiol or progesterone concentrations in the early menopause group. These findings did not demonstrate a statistically significant linear relationship (Table 6).

### 3.3. Hormonal Profile in Physiological Menopause Group (n = 181)

Descriptive statistics for the physiological menopause group are presented in Table 7. Median values were lower than mean values for most parameters, indicating skewed distributions. Hormonal variables, particularly estradiol and gonadotropins, showed wide ranges, while 25(OH)D levels demonstrated comparatively lower variability.

The correlation analysis for the physiological menopause group is presented in Table 8. Across all vitamin D subgroups, no statistically significant associations were observed between serum 25(OH)D levels and estradiol or progesterone (all *p* > 0.05). Correlation coefficients were weak and close to zero, indicating the absence of meaningful relationships between vitamin D and hormonal parameters in this group.

The correlation analysis by vitamin D subgroups for the group with physiological menopause is presented in Table 9. No statistically significant associations were observed between vitamin D and estradiol or progesterone in participants with deficient (<20 ng/mL), insufficient (21–29 ng/mL), or sufficient (≥30 ng/mL) levels (all *p* > 0.05). Correlation coefficients were weak in all subgroups.

Linear regression analysis did not reveal a significant effect of vitamin D levels on estradiol or progesterone concentrations in the group with natural menopause. These results indicate the absence of a linear relationship between vitamin D levels and reproductive hormone levels (Table 10).

## 4. Discussion

The present study was designed as a comparative observational analysis to evaluate whether consistent relationships exist between serum vitamin D levels and reproductive hormones in women with early versus physiological menopause. The overall findings were predominantly negative, with no consistent or clinically meaningful associations observed between 25(OH)D concentrations and estradiol or progesterone levels across the study population. These results are consistent with a growing body of literature reporting inconsistent or absent associations [25,26,27], and suggest that previously reported relationships may be context-dependent or influenced by unmeasured confounding factors.

As expected, the relationships between FSH and estradiol (r = −0.29, *p* = 0.0016) and LH and progesterone (r = −0.207, *p* = 0.026) seen in this study are consistent with what has been observed previously regarding the physiology of ovarian failure and indicate the expected dysregulation of hypothalamus–pituitary–ovarian relationships when women experience menopause. Our findings also support previously published studies demonstrating that the decline in ovarian hormone production often leads to compensatory rises in gonadotropin levels [8,9].

The lack of statistically significant relationships between vitamin D and reproductive hormones in the entire early menopause population supports the findings of earlier articles, which also established that there are no consistent or statistically significant correlations between vitamin D and both estradiol and progesterone levels [19,20,21]. These findings do not support a consistent association between serum vitamin D concentrations and reproductive hormone levels during menopause.

Subgroup analyses identified statistically significant correlations among participants with vitamin D levels ≥ 30 ng/mL. However, these findings were not consistent across analyses and should be interpreted cautiously due to subgroup size limitations, statistical variability, and the absence of correction for multiple comparisons. No meaningful correlations were present in the vitamin D deficient or insufficient study participants. These findings are in line with previous studies reporting inconsistent or absent correlations [10,13] and no consistent associations were identified between vitamin D status and reproductive hormone levels.

Vitamin D has been suggested to be associated with processes such as steroidogenesis, follicular development, and ovarian aging through the presence of vitamin D receptors in ovarian and hypothalamic–pituitary tissues [10,11,12,13]. However, these proposed mechanisms are primarily derived from experimental and theoretical models and remain incompletely understood in clinical populations.

In the present study, no consistent associations were observed between vitamin D levels and reproductive hormones across the study groups. Although correlations were identified in certain subgroups, these findings were not consistent and are likely influenced by sample size limitations and statistical variability.

Furthermore, no significant relationships were observed in participants with deficient or insufficient vitamin D levels in relation to estradiol or progesterone. These results suggest that any potential association between vitamin D status and reproductive hormone levels is not robust or consistently detectable within this population.

No statistically significant differences were observed between the early menopause and physiological menopause groups (36.33 ± 13.01 vs. 25.31 ± 2.68 ng/mL, *p* = 0.299) or reproductive hormone parameters, indicating substantial overlap in the endocrine profiles of the two groups. Therefore, this suggests that the early onset of menopause may represent a temporal variation in similar endocrine processes rather than a clearly distinct endocrine entity, although these findings should be interpreted cautiously, as the study design does not allow conclusions regarding underlying biological mechanisms. The observed differences in estradiol levels are consistent with expected menopausal patterns; however, no association with vitamin D status was identified, which is consistent with previous literature reporting similar hormonal patterns in menopausal women [9,24].

In addition to the similarity of vitamin D levels between groups, the high prevalence of vitamin D deficiency observed in this study is likely influenced by multiple environmental, nutritional, or lifestyle factors that were not assessed. This interpretation is consistent with the epidemiological evidence, which has shown that the high prevalence of vitamin D deficiency is common across diverse populations [11].

From a clinical perspective, although vitamin D remains important for bone health, the present study did not identify consistent associations between vitamin D status and reproductive hormone levels during menopause. Thus, these findings should be interpreted with caution given the observational design and lack of adjustment for potential confounders.

### Strengths and Limitations

This study has several strengths. It includes a moderately sized cohort of 272 women meeting predefined inclusion criteria and provides a comparative evaluation of early and physiological menopause, a distinction that remains relatively underexplored in the literature. In addition, multiple hormonal parameters were assessed, and a combination of correlation and regression analyses was applied to explore potential associations between vitamin D levels and reproductive hormones.

However, there are a number of limitations that must be taken into account. The study design precludes causal inference. Although some of the variables that could affect vitamin D levels were controlled for—including the exclusion of individuals taking vitamin D or calcium supplements, blood collection conducted over two seasons (from March to November), and the exclusion of individuals who reported extreme sun exposure—some of the other well-known confounding variables (e.g., dietary intake, physical activity, socioeconomic factors, and detailed measurement of sun exposure) were not systematically measured in this study. As a result, it is possible that the observed findings may be influenced by unmeasured confounding. In addition, serum 25(OH)D concentrations were measured using an ELISA-based method rather than LC-MS/MS, which is considered the reference analytical technique for vitamin D assessment. Therefore, analytical variability associated with immunoassay-based vitamin D measurement should also be considered when interpreting the findings. In addition, this study did not assess menopausal symptoms, bone mineral density, inflammatory biomarkers, metabolic parameters, or long-term clinical outcomes. Therefore, the findings should not be interpreted as evidence regarding broader endocrine or metabolic health consequences associated with vitamin D status during menopause.

Furthermore, the relatively small number of participants with sufficient vitamin D levels may have affected subgroup analyses and contributed to variability in the results. Furthermore, the lack of adjustment for multiple comparisons increases the risk of type I error in subgroup analyses. These limitations should be considered when interpreting the predominantly negative findings of this study.

Further prospective studies with larger samples, comprehensive assessment of confounding variables, and multivariable analytical approaches are needed to further clarify the relationship between vitamin D status and reproductive endocrinology across the menopausal transition.

## 5. Conclusions

In our study, vitamin D deficiency was highly prevalent in both early and physiological menopause. However, no consistent associations were identified between serum 25(OH)D concentrations and reproductive hormone levels across the study population. Although some subgroup-specific correlations were observed, these findings were not consistent and are likely influenced by limited sample size and statistical variability.

The results indicate a substantial overlap in hormonal profiles between early and physiological menopause, suggesting that early menopause reflects a temporal variation in similar endocrine processes rather than a distinct endocrine entity.

Overall, the findings support a cautious interpretation of previously reported associations between vitamin D status and reproductive hormone levels in menopausal women. The clinical implications of these findings remain limited, and further prospective studies with comprehensive adjustment for confounding factors are required to better understand the role of vitamin D in menopausal endocrinology.

## Figures and Tables

**Table 1:** Distribution of patients by menopause category and vitamin D status.

Menopause Category	Vitamin D <20 ng/mL	Vitamin D 21–29 ng/mL	Vitamin D ≥30 ng/mL	Total
<40 years	10	13	5	28
40–45 years	29	21	13	63
Early menopause group	39	34	18	91
46–55 years	62	62	46	170
>55 years	5	4	2	11
Physiological menopause	67	66	48	181
Total	106	100	66	272

**Table 2:** Comparative analysis of hormonal and vitamin D parameters between early and physiological menopause groups.

Parameter	Early Menopause (*n* = 91) Mean ± SD	Median (IQR)	Physiological Menopause (*n* = 181) Mean ± SD	Median (IQR)	*p*-Value
Age (years)	41.08 ± 4.38	42 (40–45)	50.35 ± 4.73	49 (46–55)	<0.001
Estradiol (pg/mL)	90.00 ± 108.66	55.89 (22.4–118.7)	72.71 ± 122.88	32.87 (14.2–85.5)	0.229
FSH (IU/L)	33.63 ± 37.41	10.93 (4.8–52.3)	44.97 ± 39.38	35.80 (18.7–67.4)	0.551
LH (mIU/mL)	18.68 ± 16.56	10.59 (5.1–28.7)	29.23 ± 23.22	22.60 (11.8–39.5)	0.274
Progesterone (ng/mL)	1.66 ± 3.75	0.22 (0.08–1.10)	0.74 ± 1.99	0.20 (0.07–0.80)	0.183
25(OH)D (ng/mL)	36.33 ± 13.01	22.23 (18.4–29.6)	25.31 ± 2.68	23.11 (19.2–28.4)	0.299

Legend: Data are presented as mean ± standard deviation and median (interquartile range, IQR). Due to non-normal and asymmetric distributions identified using the Shapiro–Wilk test, comparisons between groups were performed using the Mann–Whitney U test.

**Table 3:** Descriptive statistics in early menopause.

Parameter	Mean ± SD	Median	Min–Max
Age (years)	41.08 ± 4.38	42	34–45
Estradiol (pg/mL)	90.00 ± 108.66	55.89	5.0–712.7
FSH (mIU/mL)	33.63 ± 37.41	10.93	1.42–154.58
LH (mIU/mL)	18.68 ± 16.56	10.59	1.48–78.89
Progesterone (ng/mL)	1.66 ± 3.75	0.22	0.005–25.5
25(OH)D (ng/mL)	36.33 ± 13.01	22.23	4.75–72.9

**Table 4:** Correlation analysis in early menopause.

Variables Compared	R	*p*-Value
Estradiol vs. FSH	−0.29	0.0016
Progesterone vs. LH	−0.207	0.026
Vitamin D vs. Estradiol	−0.038	0.686
Vitamin D vs. Progesterone	0.031	0.744

**Table 5:** Correlation analysis by vitamin D subgroups for early menopause (*n* = 91).

Vitamin D Levels	Estradiol (r, *p*)	Progesterone (r, *p*)
<20 ng/mL	−0.054 (*p* = 0.697)	−0.135 (*p* = 0.339)
21–29 ng/mL	−0.056 (*p* = 0.682)	0.166 (*p* = 0.288)
≥30 ng/mL	−0.780 (*p* = 0.0016)	0.534 (*p* = 0.0104)

**Table 6:** Linear regression analysis for early menopause group.

Dependent Variable	Slope	95% CI	*p*-Value	R^2^
Estradiol	0.368	−4.233 to 4.968	0.875	0.00022
Progesterone	0.089	−0.069 to 0.247	0.265	0.011

**Table 7:** Descriptive statistics in the physiological menopause group (*n* = 181).

Parameter	Mean ± SD	Median	Min–Max
Estradiol (pg/mL)	50.35 ± 4.73	49	46–55
FSH (mIU/mL)	72.71 ± 122.88	32.87	5.0–584.8
LH (mIU/mL)	44.97 ± 39.38	35.80	1.01–200.0
Progesterone (ng/mL)	29.23 ± 23.22	22.60	1.48–142.0
25(OH)D (ng/mL)	0.74 ± 1.99	0.20	0.03–25.5
Estradiol (pg/mL)	25.31 ± 2.68	23.11	3.0–59.1

**Table 8:** Correlation analysis in the physiological menopause group.

Variables Compared	R	*p*-Value
Vitamin D (<20 ng/mL) vs. Estradiol	−0.054	0.697
Vitamin D (<20 ng/mL) vs. Progesterone	−0.135	0.339
Vitamin D (21–29 ng/mL) vs. Estradiol	−0.056	0.682
Vitamin D (21–29 ng/mL) vs. Progesterone	0.023	0.869
Vitamin D (≥30 ng/mL) vs. Estradiol	−0.141	0.373
Vitamin D (≥30 ng/mL) vs. Progesterone	−0.250	0.106

**Table 9:** Correlation analysis by vitamin D subgroups for the physiological menopause group (*n* = 181).

Vitamin D Levels	Estradiol (r, *p*)	Progesterone (r, *p*)
<20 ng/mL	−0.054 (*p* = 0.697)	−0.135 (*p* = 0.339)
21–29 ng/mL	−0.056 (*p* = 0.682)	0.023 (*p* = 0.869)
≥30 ng/mL	−0.141 (*p* = 0.373)	−0.250 (*p* = 0.106)

**Table 10:** Linear regression analysis for the physiological menopause group.

Dependent Variable	Slope	95% CI	*p*-Value	R^2^
Estradiol	−0.076	−0.24 to 0.09	0.352	0.02
Progesterone	0.067	−0.07 to 0.20	0.351	0.02

## Data Availability

The data presented in this study are available on reasonable request from the corresponding author.

## Abbreviations

The following abbreviations are used in this manuscript:

25(OH)D	25-Hydroxyvitamin D
ANOVA	Analysis of Variance
BMI	Body Mass Index
CI	Confidence Interval
ELISA	Enzyme-Linked Immunosorbent Assay
FSH	Follicle-Stimulating Hormone
IQR	Interquartile Range
LC-MS/MS	Liquid Chromatography–Tandem Mass Spectrometry
LH	Luteinizing Hormone
LOB	Limit of Blank
LOD	Limit of Detection
POI	Premature Ovarian Insufficiency
R^2^	Coefficient of Determination
SD	Standard Deviation
VIF	Variance Inflation Factor
